# Lipoprotein combine index is associated with non-alcoholic fatty liver disease: a 5-year longitudinal cohort study in non-obese Chinese populations with normal lipids

**DOI:** 10.3389/fmed.2025.1618576

**Published:** 2025-08-15

**Authors:** Weitao Wu, Donghai Wu, Changchun Cao, Ronghua Zhou, Shihua Ding, Ying Ying, Dayong Sun, Haofei Hu

**Affiliations:** ^1^Department of Gastroenterology, Shenzhen Second People’s Hospital, The First Affiliated Hospital of Shenzhen University, Shenzhen, Guangdong, China; ^2^Shenzhen University Health Science Center, Shenzhen, Guangdong, China; ^3^Department of Rehabilitation, Shenzhen Dapeng New District Nan’ao People’s Hospital, Shenzhen, Guangdong, China; ^4^Department of Endoscopy, Pengpai Memorial Hospital, Shanwei, Guangdong, China; ^5^Department of Physiology, Shenzhen University Medical School, Shenzhen, Guangdong, China; ^6^Department of Nephrology, Shenzhen Second People’s Hospital, The First Affiliated Hospital of Shenzhen University, Shenzhen, Guangdong, China

**Keywords:** non-alcoholic fatty liver disease, lipoprotein combine index, cohort study, Cox proportional-hazards regression, non-linear

## Abstract

**Objective:**

Current evidence concerning the association between lipoprotein combine index (LCI) and Non-alcoholic fatty liver disease (NAFLD) in non-obese people remains limited. This 5-year longitudinal cohort study aimed to explore the connection between LCI and risk of NAFLD in non-obese Chinese individuals with normal lipids.

**Methods:**

This retrospective cohort study from January 2010 to December 2014 consecutively and non-selectively collected 9,838 non-obese participants with normal lipid profiles in a Chinese hospital. Using the Cox proportional-hazards regression model, we explored the relationship between baseline LCI and NAFLD risk. We applied cubic spline functions and curve fitting to characterize the non-linear association between LCI and NAFLD. Simultaneously, we conducted sensitivity and subgroup analyses, and employed receiver operating characteristic (ROC) curve analysis to evaluate the predictive potential of LCI for NAFLD incidence.

**Results:**

The mean age of participants was 42.46 ± 14.70 years, with males comprising 51.40% of the cohort. During a median follow-up period of 33.10 months, 855 participants (8.89%) progressed NAFLD, with an incidence of 31.51 cases per 1,000 person-years. A significant non-linear relationship was identified between LCI and NAFLD risk with an inflection point at 5.514 mmol^2^/L^2^, where the HR was significantly stronger below this threshold (HR = 1.282, 95%CI: 1.162–1.415) compared to above it (HR = 1.063, 95%CI: 1.042–1.084). Subgroup analysis revealed the strongest associations in participants with body mass index (BMI) between 18.5 and 24 kg/m^2^. LCI demonstrated superior predictive value for NAFLD compared to individual lipid parameters, with an area under the ROC curve of 0.717.

**Conclusion:**

This study offers novel insights into the relationship between LCI and NAFLD risk in non-obese Chinese individuals with normal lipid levels. The non-linear association and the moderate discriminatory ability of LCI suggest its potential utility as a practical screening marker for population-level risk stratification and early preventive strategies in seemingly low-risk, normal-weight populations.

## Background

Non-alcoholic fatty liver disease (NAFLD) is characterized by a spectrum of liver injuries resulting from abnormal lipid metabolism and hepatic fat accumulation (1, 2). With the increasing prevalence of metabolic disorders, NAFLD has emerged as the leading cause of chronic liver disease worldwide, with global prevalence reaching 38% (3, 4). Notably, the incidence of NAFLD in non-obese individuals has been steadily rising, now accounting for approximately 40% of NAFLD cases (5). This subgroup presents a particularly complex clinical scenario, with emerging evidence suggesting that non-obese NAFLD patients may experience more aggressive disease progression and potentially more severe clinical outcomes compared to their obese counterparts (6).

The pathogenetic mechanisms underlying NAFLD reveal an intricate interplay of metabolic processes (1, 7). While abnormal blood lipids have long been considered an important pathogenic factor (8, 9), emerging research indicates that many non-obese individuals with NAFLD actually maintain normal lipid levels (10, 11). Given the increasing prevalence and complexity of non-obese NAFLD, continued investigation is essential to identify novel lipid metabolism risk factors for individuals with normal lipid levels, thereby improving more precise preventive, diagnostic, and therapeutic strategies.

Recent research suggests the Lipid Composite Index (LCI) effectively predicts metabolic and cardiovascular risks with greater accuracy than individual lipid measurements alone (12–15). Furthermore, a cross-sectional investigation conducted in a Japanese population demonstrated a significant positive association between LCI and NAFLD, revealed LCI as a more reliable marker for NAFLD identification than traditional lipid parameters (16). However, longitudinal evidence examining the relationship between baseline LCI and NAFLD development remains limited, especially in non-obese Chinese within normal lipids profile. Therefore, this 5-year longitudinal cohort study aims to investigate whether LCI can effectively predict NAFLD incidence in this unique population, potentially providing clinicians with a valuable tool for risk stratification and early preventive strategies in non-obese individuals with normal lipid profiles who may nevertheless be at risk for metabolic complications such as NAFLD.

## Materials and methods

### Study design

This study utilized a longitudinal cohort design, drawing on data from the electronic health records of Wenzhou People’s Hospital in China. The primary independent variable analyzed was LCI, with the outcome variable being the presence of NAFLD, categorized as a binary variable (0 = non-NAFLD, 1 = NAFLD).

### Data source

The raw data was freely accessed from the DATADRYAD database^1^ under the doi: 10.5061/dryad.1n6c4.14, contributed by Sun, et al. (10). According to Dryad’s terms of service, researchers are permitted to use this data for secondary analyses without infringing on the authors’ rights.

### Study participants

To mitigate selection bias, the research team systematically enrolled consecutive participants from health examinations at Wenzhou Medical Center. Their identities were encoded with non-traceable codes to ensure privacy. The ethics committee of Wenzhou People’s Hospital approved this study, and all participants provided informed consent to participate (10). All methods complied with the relevant guidelines and regulations, as stated in the Declarations section.

**FIGURE 1:**
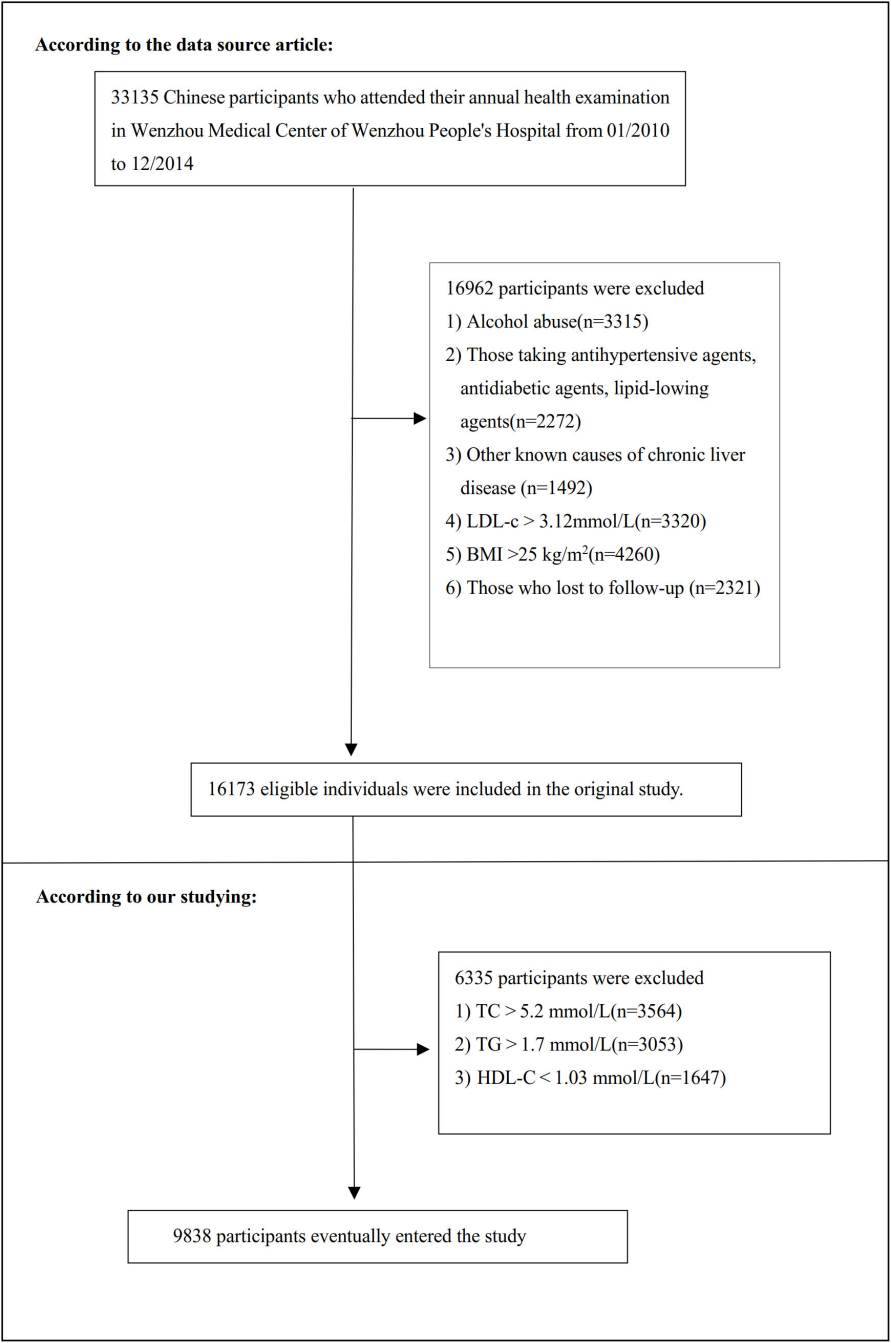
Flowchart of study participants. Flowchart illustrated the systematic screening from 33,135 potential participants to the final cohort of 9,838 non-obese Chinese individuals with normal lipid profiles.

The research initially screened 33,135 potential participants, with 16,997 subsequently excluded through rigorous selection criteria. The final analysis encompassed 9,838 participants (detailed in Figure 1). The baseline clinical data collection spanned from January 2010 to December 2014. Inclusion criteria included: NAFLD-free Chinese individuals in the longitudinal studies who participated in a health examination from January 2010 to December 2014. The exclusion criteria the initial investigation included (10): (1) excessive alcohol consumption (≥ 140 g/week for males and ≥ 70 g/week for females); (2) known causes of chronic hepatic diseases, such as NAFLD, autoimmune hepatitis, or viral hepatitis; (3) BMI ≥ 25 kg/m^2^; (4) LDL-c > 3.12 mmol/L (17); (5) use of antihypertensive, lipid-lowering, or anti-diabetic medications; and (6) those who lost to follow-up. Furthermore, our current analysis excluded participants with additional lipid levels exceeding established thresholds (TC > 5.2 mmol/L, TG > 1.7 mmol/L or HDL-c < 1.03 mmol/L) (17).

### Variables

#### Lipoprotein combine index

At baseline, LCI was calculated as TC × TG × LDL-C/HDL-c according to established methodology (TC, Total cholesterol; TG, Triglyceride; LDL-c, low-density lipoprotein cholesterol; HDL-c, high-density lipoprotein cholesterol) (18, 19). All lipid parameters (TC, TG, LDL-c, and HDL-c) were measured in mmol/L, with the resulting LCI expressed in mmol^2^/L^2^. LCI was analyzed as a continuous variable.

#### Outcome measures

The primary outcome variable of interest was NAFLD, classified as a dichotomous variable (0 = non-NAFLD, 1 = NAFLD). The process for diagnosing NAFLD was as follows: participants were evaluated using ultrasonography, in accordance with the guidelines set forth by the Chinese Liver Disease Association (20). Specifically, five criteria were employed for the diagnosis of NAFLD: (1) Diffusely enhanced near-field echoes in the liver area, with progressively attenuated far-field echoes; (2) Unclear intrahepatic cavity structure; (3) Mild to moderate hepatomegaly with rounded edges; (4) Decreased blood flow signals in the liver; (5) Poor visualization or incompleteness of the right hepatic lobe and diaphragmatic capsule (10).

Annual follow-up assessments were conducted throughout the observation period. Liver ultrasonography, performed in a blinded manner (similar to the baseline assessment), was utilized to determine the incidence of NAFLD. Participants were censored at the time of NAFLD diagnosis or at their last visit, whichever occurred first. The total follow-up period lasted for 5 years.

#### Covariates

In our study, covariates were selected based on our clinical experience and existing literature (10, 21). Accordingly, the following variables were identified as covariates: (1) continuous variables: age, body mass index (BMI), systolic blood pressure (SBP), alanine aminotransferase (ALT), diastolic blood pressure (DBP), albumin (ALB), aspartate aminotransferase (AST), globulin (GLB), γ-glutamyl transpeptidase (GGT), direct bilirubin (DBIL), alkaline phosphatase (ALP), uric acid (UA), total bilirubin (TB), blood urea nitrogen (BUN), serum creatinine (Scr) and fasting plasma glucose (FPG); (2) categorical variables: gender.

All biochemical values were measured using standard methods by an automated analyzer (Abbott AxSYM). A physician conducted a health habit inventory and collected the medical history (10). BMI was calculated as weight in kilograms divided by height in square meters (kg/m^2^). Data were collected under standardized conditions and processed uniformly. According to the World Health Organization (WHO), impaired fasting glucose (IFG) was defined as FPG level between 6.1 and 6.9 mmol/L, FPG ≥ 7 mmol/L was defined as diabetes (22). ALT > 40 U/L reflected liver dysfunction. More specific details were presented in the previous reports (21).

### Statistical analysis

All analyses were conducted using the statistical software packages R (The R Foundation)^2^ and EmpowerStats (X&Y Solutions, Inc., Boston, MA)^3^.

Participants were stratified based on quartiles of LCI. Continuous variables are expressed as mean (standard deviation) for normally distributed data or median (range) for non-normally distributed data, while categorical variables are presented as counts (percentages). We utilized the One-Way ANOVA test for normally distributed data, the chi-squared (χ^2^) test for categorical variables, and the Kruskal-Wallis H test for skewed distributions to assess differences among various LCI groups.

The collinearity of covariates was evaluated using the variance inflation factor (VIF), calculated as VIF = 1/(1-R^2^). Here, R^2^ refers to the R-squared value obtained from a linear regression equation, where the dependent variable is the variable of interest and the independent variables include all other variables. Variables with a VIF greater than 5 are considered collinear and cannot be included in the multiple regression model (Supplementary Table 1).

To investigate the association between LCI and NAFLD, we developed a comprehensive Cox proportional-hazards regression approach after collinearity screening. Our methodology comprised three distinct models: Crude Model: A baseline assessment with no covariate adjustments; Model I: Minimal adjustment of sociodemographic variables (age, gender, SBP, DBP, and BMI); Model II: Full adjustment of covariates from Table 1, including age, SBP, sex, DBP, AST, BMI, ALB, GGT, GLB, ALP, DBIL, BUN, ALT, FBG, TB and UA. We recorded effect sizes using hazard ratios (HR) with 95% confidence intervals (CI). The analysis was informed by preliminary collinearity screening results.

**TABLE 1:** The baseline characteristics of participants.

Variable	Q1 (< 3.67)	Q2 (3.67–5.67)	Q3 (5.67–8.53)	Q4 (≥ 8.53)	*P*-value
Participants	2460	2459	2459	2460	
Age (years)	41.65 ± 14.41	42.34 ± 14.66	42.84 ± 14.99	43.00 ± 14.71	0.005
BMI (kg/m^2^)	20.34 ± 1.94	20.67 ± 2.01	21.22 ± 1.99	21.89 ± 1.89	<0.001
SBP (mmHg)	114.30 ± 15.03	116.31 ± 15.88	119.31 ± 16.16	122.39 ± 16.11	<0.001
DBP (mmHg)	68.95 ± 9.21	70.13 ± 9.72	72.13 ± 10.06	73.97 ± 10.08	<0.001
ALP (U/L)	63.75 ± 21.31	67.28 ± 24.67	69.56 ± 21.19	73.49 ± 21.63	<0.001
GGT (U/L)	17.00 (13.00–24.00)	18.00 (14.00–26.00)	20.00 (15.00–29.50)	23.00 (17.00–33.00)	<0.001
ALT (U/L)	14.00 (10.00–21.00)	15.00 (11.00–22.00)	16.00 (11.00–22.00)	17.00 (12.00–25.00)	<0.001
AST (U/L)	21.72 ± 10.15	21.76 ± 8.45	22.05 ± 8.71	22.64 ± 8.60	<0.001
ALB (g/L)	44.11 ± 2.76	44.17 ± 2.75	44.41 ± 2.65	44.42 ± 2.85	<0.001
TB (μmol/L)	12.14 ± 5.08	12.02 ± 4.98	12.13 ± 4.91	12.35 ± 4.81	0.125
DBIL (μmol/L)	2.30 (1.70–3.10)	2.20 (1.60–3.00)	2.20 (1.60–3.00)	2.10 (1.40–2.90)	<0.001
BUN (mmol/L)	4.39 ± 1.32	4.45 ± 1.46	4.47 ± 1.21	4.56 ± 1.34	<0.001
Scr (μmol/L)	72.00 ± 21.26	75.59 ± 34.86	77.90 ± 18.50	82.68 ± 22.52	<0.001
UA (μmol/L)	237.53 ± 74.21	253.97 ± 77.23	270.42 ± 77.43	293.71 ± 79.76	<0.001
FBG (mmol/L)	4.95 ± 0.64	5.03 ± 0.65	5.10 ± 0.61	5.21 ± 0.86	<0.001
TC (mmol/L)	3.95 ± 0.55	4.29 ± 0.48	4.46 ± 0.44	4.67 ± 0.36	<0.001
TG (mmol/L)	0.68 ± 0.18	0.85 ± 0.19	1.04 ± 0.21	1.31 ± 0.22	<0.001
HDL-c (mmol/L)	1.71 ± 0.32	1.57 ± 0.28	1.46 ± 0.24	1.31 ± 0.18	<0.001
LDL-c (mmol/L)	1.70 ± 0.34	2.05 ± 0.29	2.24 ± 0.30	2.48 ± 0.28	<0.001
**Gender**					<0.001
Female	1270 (51.63%)	1240 (50.43%)	1140 (46.36%)	1131 (45.98%)	
Male	1190 (48.37%)	1219 (49.57%)	1319 (53.64%)	1329 (54.02%)	

Values are *n* (%) or mean ± SD or median (quartile). BMI, body mass index; DBP, diastolic blood pressure; ALP, alkaline phosphatase; SBP, systolic blood pressure; GGT, γ-glutamyl transpeptidase; AST, aspartate aminotransferase; TG, Triglyceride; ALB, albumin; ALT, alanine aminotransferase; GLB, globulin; LDL-c, low-density lipid cholesterol; BUN, Serum urea nitrogen; HDL-c, high-density lipoprotein cholesterol; Scr, serum creatinine; TC, Total cholesterol; FPG, fasting plasma glucose; UA, uric acid; DBIL, direct bilirubin; TB, total bilirubin; LCI, lipoprotein combine index.

To address the non-linear relationship between LCI and NAFLD, we applied Cox proportional hazards regression model with cubic spline functions and the smooth curve fitting to address non-linearity. In addition, a two-piecewise Cox proportional hazards regression model was employed to provide a deeper explanation of the non-linear relationship between LCI and NAFLD (23). A log-likelihood ratio test was conducted to identify the most appropriate model for describing the LCI-NAFLD risk relationship.

We conducted comprehensive subgroup analyses using stratified Cox proportional-hazards regression to explore potential effect modifiers. Our approach involved converting continuous variables to clinically relevant categories (age groups: < 30, 30–40, 40–50, 50–60, ≥ 60 years; BMI: < 18.5, 18.5–24, ≥ 24 kg/m^2^) and standardizing clinical parameters (FPG, ALT, AST, ALP, GGT, UA, SBP, DBP, eGFR) to established cut-points (21). Beyond stratification factors, we rigorously controlled for multiple potential confounders. We employed likelihood ratio tests to detect significant interaction effects, ensuring a comprehensive and nuanced analysis of potential modifying factors (24). And eGFR was calculated using the CKD-EPI formula specific to Asian populations (25).

The number of participants with missing data of ALP, GGT, ALT, AST, ALB, GLB, TB, DBIL, SBP, DBP, FBP, UA, Scr, and BUN were 2563 (26.05%), 2565 (26.07%), 2563 (26.05%), 2563 (26.05%), 879 (8.93%), 879 (8.93%), 3491 (35.48%), 4405 (44.78%), 9 (0.09%), 9 (0.09%), 1 (0.01%), 1 (0.01%), 1 (0.01%) and 1 (0.01%) respectively. To mitigate potential bias, multiple imputation techniques were employed under the missing-at-random (MAR) assumption (26). The comprehensive imputation model incorporated age, sex, BMI, AST, SBP, ALB, ALT, ALP, DBP, GLB, HDL-c, DBIL, BUN, TG, UA, GGT, FBG, TC, TB, and LDL-c. To address missing data, we employed multiple imputation using the mice software package, generating five complete datasets through chained equations. Sensitivity analysis was conducted to assess potential differences between imputed and original data (Supplementary Table 3). Final results of our study were pooled according to Rubin’s rules (27).

To validate result reliability, multiple sensitivity analyses were conducted. LCI was transformed into a quartile-based categorical variable, with trend analysis performed to examine potential non-linear relationships. Considering established risk factors for NAFLD, subsequent analyses strategically excluded participants with specific metabolic and liver function abnormalities: FPG > 6.1 mmol/L, TG ≥ 1.7 mmol/L, eGFR <60 mL/min⋅1.73 m^2^, ALB <35 g/L and ALT > 40 U/L (2, 21). A generalized additive model (GAM) was applied to evaluate covariate continuity, and E-values were calculated to assess potential unmeasured confounding between LCI and NAFLD risk (28).

Following the association results, we evaluated the efficacy of LCI against traditional lipid parameters in predicting the occurrence of NAFLD via ROC analysis, with area under the curve (AUC) comparisons performed using the DeLong methodology (29). Results were documented in strict adherence to the STROBE statement (30). *P* values less than 0.05 (two-sided) were considered statistically significant.

## Results

### Baseline characteristics of participants

Baseline participant characteristics (Table 1) revealed a cohort with mean age 42.46 ± 14.70 years, 51.40% male, and median LCI of 5.67 (3.67–8.53). During 33.10 months of follow-up, 8.89% developed NAFLD. Participants were stratified into LCI quartiles, with Q4 (≥ 8.53) showing significant metabolic and biochemical divergences compared to Q1 (< 3.67). In Q4 compared to Q1, participants showed significant increases in male, age, BMI, SBP, DBP, ALP, GGT, ALT, AST, ALB, BUN, Scr, UA, FBG, TG, TC, LDL-c, while female participants and markers like DBIL, HDL-c exhibited opposite trends.

**FIGURE 2:**
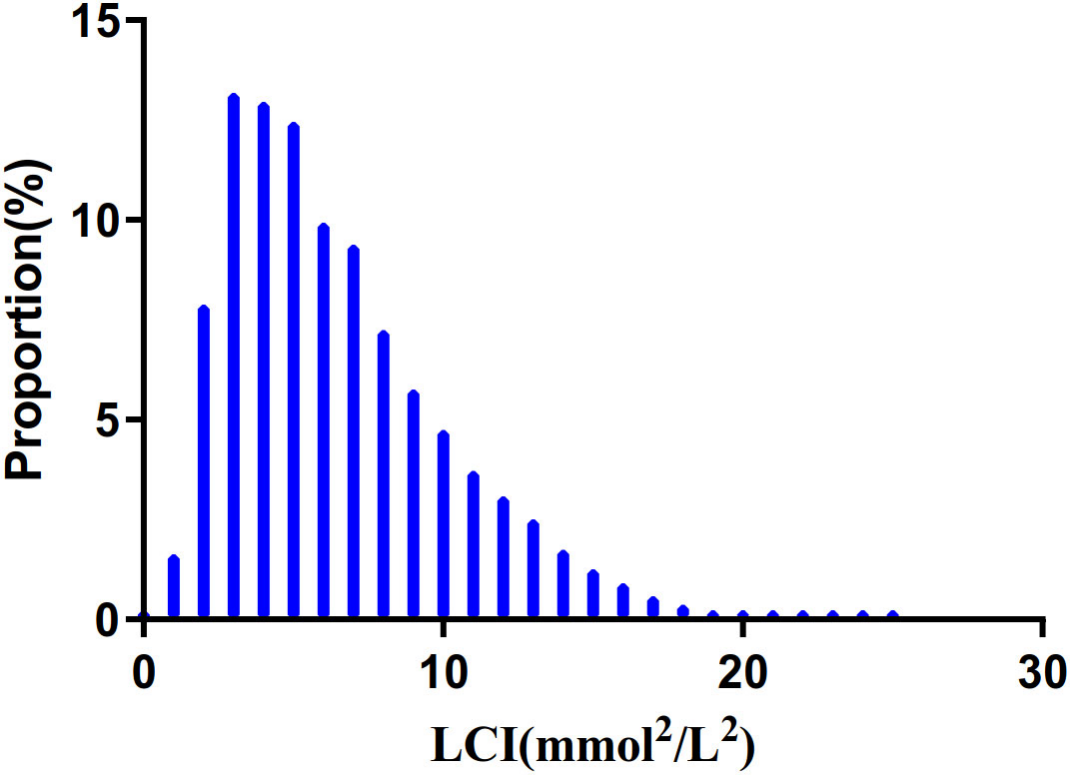
Distribution of LCI. It presented a non-normal LCI distribution while being in the range from 0.302 to 24.493 (mmol^2^/mL^2^), with an average of 6.503 (mmol^2^/mL^2^). LCI, lipoprotein combine index.

**FIGURE 3:**
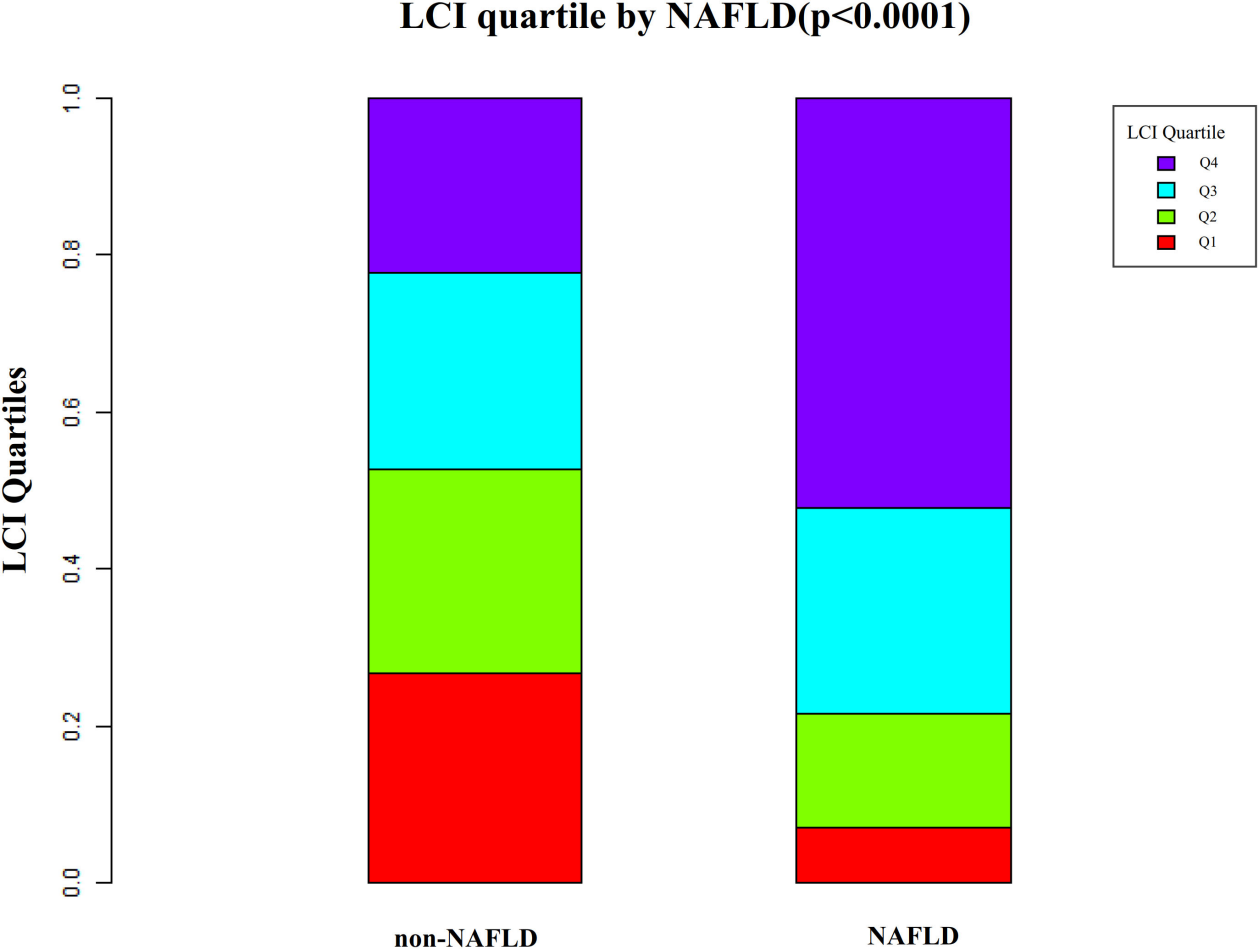
Data visualization of LCI of all participants from the NAFLD and non-NAFLD groups. It indicated that the distribution level of LCI in the NAFLD group was lower. In contrast, the LCI level in the non-NAFLD group was relatively higher. LCI, lipoprotein combine index; NAFLD, non-alcoholic fatty liver disease.

**FIGURE 4:**
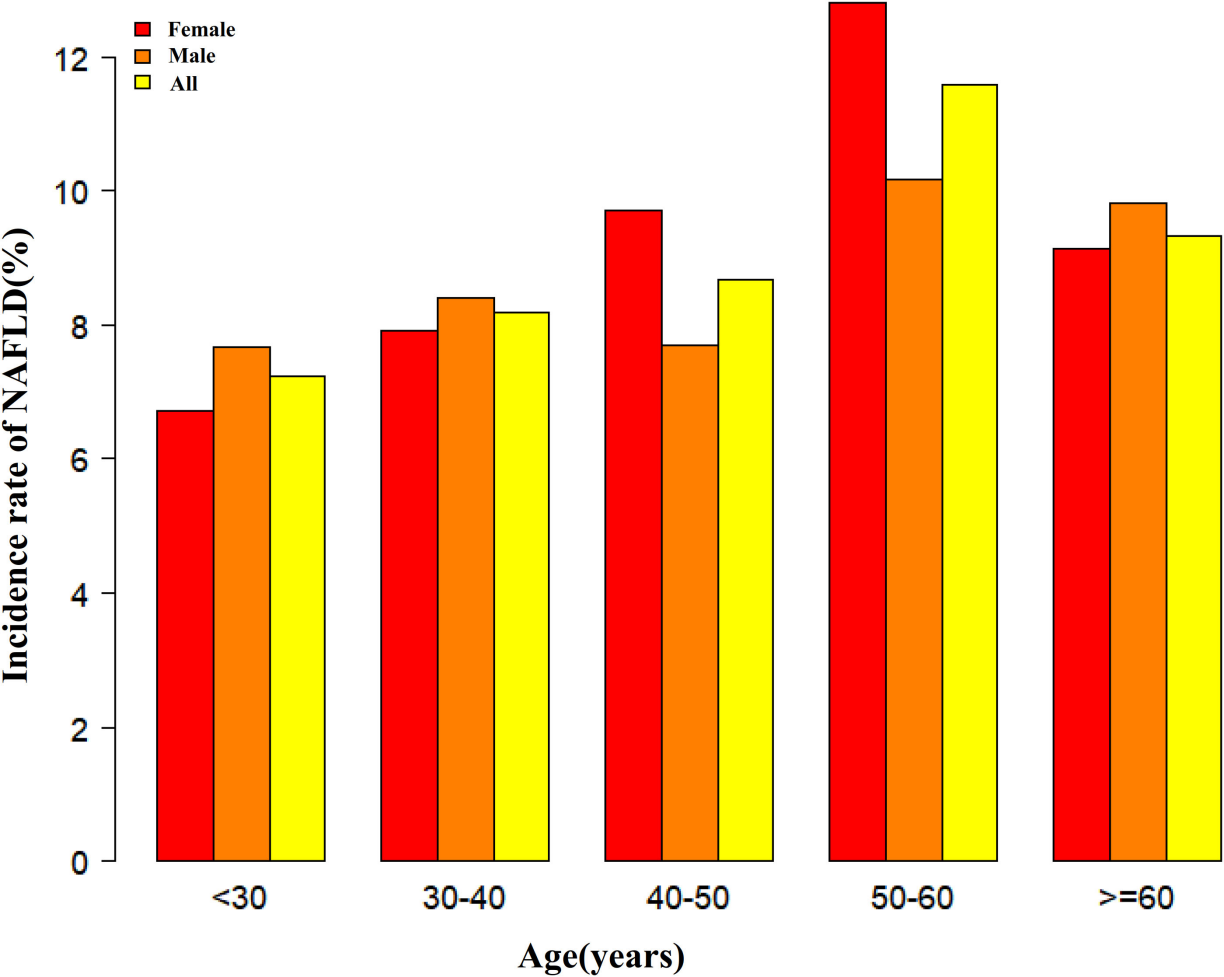
NAFLD incidence rate of age stratification by 10 intervals. It showed that in age stratification by 10 intervals, except for age >60, male participants had a higher incidence of NAFLD than female participants no matter what age group they were in. It also found that the incidence of NAFLD increased with age, both in males (except for age >60 years) and females (except 40–50 years old) participants. NAFLD, non-alcoholic fatty liver disease.

Figure 2 depicts LCI distribution, ranging from 0.30 to 24.49 mmol^2^/L^2^ with a non-normal pattern, mean of 6.50 mmol^2^/L^2^ and median of 5.67 mmol^2^/L^2^. Comparative analysis between NAFLD and non-NAFLD groups (Figure 3) revealed notably higher LCI levels in the NAFLD cohort. Age-stratified analysis (Figure 4) demonstrated gender-specific NAFLD incidence patterns: males consistently showed higher NAFLD rates across most age groups, with the exception of those ≥ 60 years. Notably, NAFLD incidence progressively increased with age among non-elderly participants in both genders (excluding the 40–50 years interval).

### The incidence rate of NALFD

Table 2 revealed that during a median 33.10-month follow-up, 855 (8.69%) participants developed NAFLD, with a total cumulative incidence of 31.51 per 1000 person-years. LCI quartile-specific cumulative incidences were 8.68, 18.34, 33.22, and 66.99 per 1000 person-years, respectively. NAFLD incidence rates across groups were 2.44% (1.83%–3.05%), 5.08% (4.21%–5.95%), 9.11% (7.97%–10.25%), and 18.13% (16.61%–19.65%), demonstrating a significant trend of increasing NAFLD risk with higher LCI (*p* < 0.0001).

**TABLE 2:** Incidence rate of incident NAFLD.

LCI	Participants (*n*)	NAFLD events (*n*)	Incidence rate (95%CI) (%)	Per 1000 person-year
Total	9838	855	8.69 (8.13–9.25)	31.51
Q1 (< 82.46)	2460	60	2.44 (1.83–3.05)	8.68
Q2 (82.46–99.33)	2459	125	5.08 (4.21–5.95)	18.34
Q3 (99.33–116.33)	2459	224	9.11 (7.97–10.25)	33.22
Q4 (≥ 116.33)	2460	446	18.13 (16.61–19.65)	66.99
P for trend			<0.001	

LCI, lipoprotein combine index (mmol^2^/L^2^); NAFLD, non-alcoholic fatty liver disease.

**FIGURE 5:**
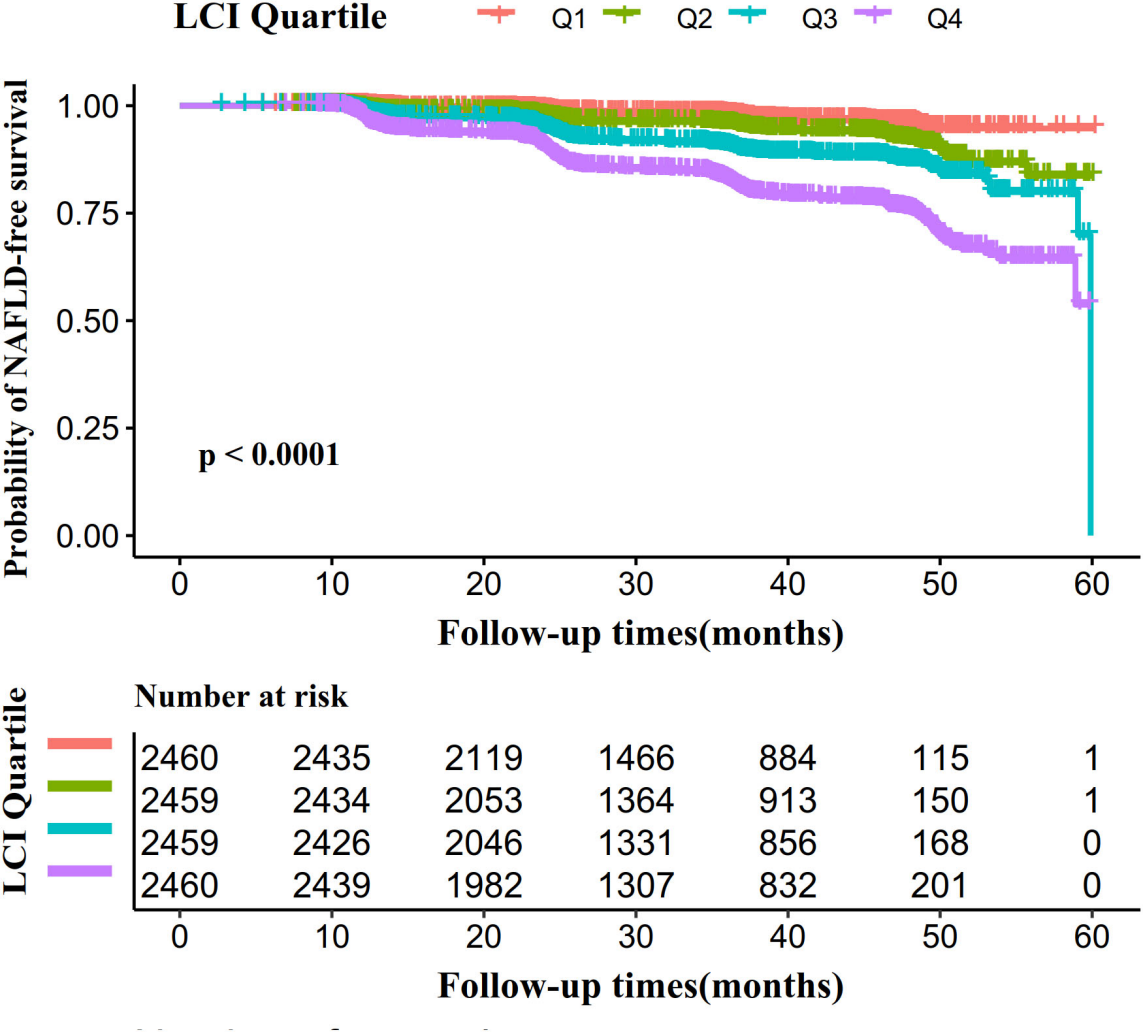
Kaplan–Meier event-free survival curve. The probability of NAFLD-free survival differed significantly between LCI groups (log-rank test, *p* < 0.0001). The probability of NAFLD-free survival gradually decreased with increasing LCI, suggesting that the group with the highest LCI had the highest risk of NAFLD. LCI, lipoprotein combine index; NAFLD, non-alcoholic fatty liver disease.

Figure 5 presents Kaplan–Meier survival curves illustrating NAFLD-free survival probability across LCI groups. Statistical analysis revealed significantly different NAFLD-free survival chances among groups (log-rank test, *P* < 0.001). The Q4 group (LCI ≥ 8.53) demonstrated the highest NAFLD risk, with NAFLD-free survival probability progressively declining as LCI increased.

### The association between LCI and NAFLD

**FIGURE 6:**
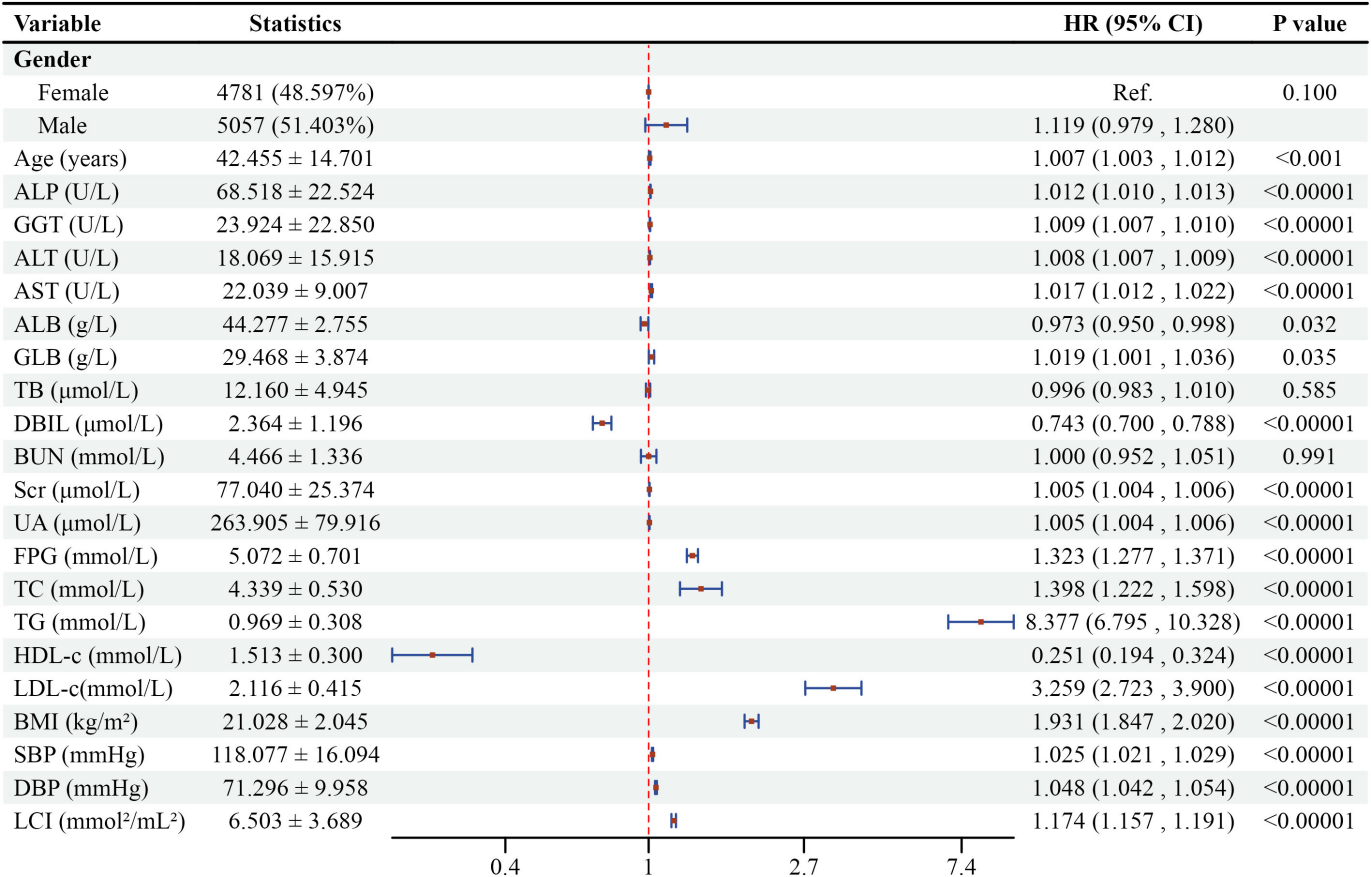
Forest plot of univariate Cox proportional hazards model for NAFLD risk factors. Forest plot showing hazard ratios (HR) and 95% confidence intervals (CI) from univariate Cox regression analysis for factors associated with NAFLD development.

Figure 6 revealed univariate analysis results showing multiple factors positively correlated with NAFLD: male sex, age, ALP, GGT, ALT, AST, GLB, Scr, UA, FPG, TC, TG, LDL-c, BMI, SBP, DBP, and LCI. Conversely, ALB, DBIL, and HDL-c demonstrated negative correlations with NAFLD. TB and BUN showed no significant association with NAFLD (all *P* > 0.05).

Table 3 demonstrated the association between LCI and NAFLD risk using multivariate Cox proportional-hazards regression models. In Model 1, each 1-unit (mmol2/L2) LCI increase corresponded to a 17.4% higher NAFLD risk (HR = 1.174, 95% CI: 1.157–1.191, *P* < 0.0001). After demographic adjustments in Model 2, the NAFLD risk increased by 10.7% per LCI unit (HR = 1.107, 95% CI: 1.090–1.124, *P* < 0.0001). Model 3, adjusting for comprehensive factors, showed an 8.5% NAFLD risk increase per LCI unit (HR = 1.085, 95% CI: 1.068–1.102, *P* < 0.0001). The Q4 group (LCI ≥ 8.53) consistently demonstrated significantly elevated NAFLD risk across all models compared to the lowest quartile: Model 1 (HR = 7.597, 95% CI: 5.801–9.949, *P* < 0.001), Model 2 (HR = 3.720, 95% CI: 2.832–4.885, *P* < 0.0001), and Model 3 (HR = 3.022, 95% CI: 2.288–3.991, *P* < 0.0001).

**TABLE 3:** Relationship between LCI and the incident NAFLD in different models.

Exposure	Crude model (HR,95%CI, P)	Model I (HR,95%CI, P)	Model II (HR,95%CI, P)	Model III (HR,95%CI, P)
LCI	1.174 (1.157, 1.191) < 0.00001	1.107 (1.090, 1.124) < 0.00001	1.085 (1.068, 1.102) < 0.00001	1.073 (1.056, 1.091) < 0.00001
**LCI Quartile**				
Q1	Ref.	Ref.	Ref.	Ref.
Q2	2.091 (1.537, 2.845) < 0.00001	1.737 (1.277, 2.365) 0.00044	1.672 (1.226, 2.282) 0.00118	1.542 (1.129, 2.104) 0.00642
Q3	3.765 (2.831, 5.007) < 0.00001	2.393 (1.797, 3.188) < 0.00001	2.209 (1.652, 2.954) < 0.00001	1.917 (1.432, 2.568) 0.00001
Q4	7.597 (5.801, 9.949) < 0.00001	3.720 (2.832, 4.885) < 0.00001	3.022 (2.288, 3.991) < 0.00001	2.530 (1.912, 3.349) < 0.00001
P for trend	<0.00001	<0.00001	<0.00001	<0.00001

Crude model: we did not adjust other covariates. Model I: we adjusted age, sex, BMI, SBP, DBP. Model II: we adjusted age, SBP, sex, ALT, BMI, GGT, DBP, ALP, ALB, GLB, DBIL, AST, TB, UA, FBG and BUN. Model III: we adjusted sex and incorporated smoothed transformations for continuous variables in Model II. HR, hazard ratios; CI, confidence; Ref, reference; LCI, lipoprotein combine index (mmol^2^/L^2^); NAFLD, non-alcoholic fatty liver disease.

### Sensitivity analysis

Sensitivity analyses were conducted to verify the robustness of the findings. In Model 4, a Generalized Additive Model (GAM) with a continuous covariate was employed, showing a consistent association (HR = 1.073, 95% CI: 1.056–1.091, *P* < 0.0001) (Table 3). Further sensitivity analyses excluded patients with FPG > 6.1 mmol/L, ALT > 40 U/L, ALB <35 g/L and eGFR <60 mL/min⋅1.73 m^2^. The results in Table 4 confirmed the robustness of the association between the LCI and NAFLD. The E-value of 1.39 suggests that unmeasured confounders would minimally impact the relationship between LCI and incident NAFLD. This indicates the robustness of our findings and the limited potential for alternative explanations to significantly alter our results. To ensure reliable findings, sensitivity analysis was conducted comparing results from pre-imputation data with five imputed datasets, demonstrating robust associations between LCI and NAFLD ((Supplementary Table 3).

**TABLE 4:** Relationship between LCI and NAFLD in different sensitivity analyses.

Exposure	Model I (HR,95%CI, P)	Model II (HR,95%CI, P)	Model III (HR,95%CI, P)	Model IV (HR,95%CI, P)
LCI	1.088 (1.069, 1.107) < 0.00001	1.080 (1.063, 1.098) < 0.00001	1.085 (1.068, 1.102) < 0.00001	1.085 (1.067, 1.104) < 0.00001
**LCI (Quartile)**				
Q1	Ref.	Ref.	Ref.	Ref.
Q2	1.693 (1.212, 2.365) 0.00202	1.730 (1.255, 2.385) 0.00082	1.675 (1.227, 2.285) 0.00115	1.787 (1.285, 2.484) 0.00056
Q3	2.126 (1.551, 2.914) < 0.00001	2.107 (1.557, 2.852) < 0.00001	2.209 (1.652, 2.955) < 0.00001	2.263 (1.660, 3.086) < 0.00001
Q4	3.037 (2.246, 4.106) < 0.00001	2.954 (2.211, 3.946) < 0.00001	3.026 (2.292, 3.997) < 0.00001	3.143 (2.331, 4.236) < 0.00001
P for trend	<0.00001	<0.00001	<0.00001	<0.00001

Model I was sensitivity analysis in participants without FPG > 6.1 mmol/L (*N* = 9412). We adjusted age, SBP, sex, ALT, BMI, GGT, DBP, ALP, ALB, GLB, DBIL, AST, TB, UA, FBG, BUN. Model II was sensitivity analysis in participants without ALT > 40 U/L (*N* = 9367). We adjusted the same covariates as Model I. Model III was sensitivity analysis in participants without ALB <35 g/L mmol/L (*N* = 9814). We adjusted the same covariates as Model I. Model IV was sensitivity analysis in participants without eGFR <60 mL/min⋅1.73 m^2^ (*N* = 9397). We adjusted he same covariates as Model I. HR, Hazard ratios; CI, confidence; Ref, reference; eGFR, evaluated glomerular filtration rate; LCI, Lipoprotein combine index (mmol^2^/L^2^); NAFLD, non-alcoholic fatty liver disease.

### The non-linearity between LCI and NAFLD

**FIGURE 7:**
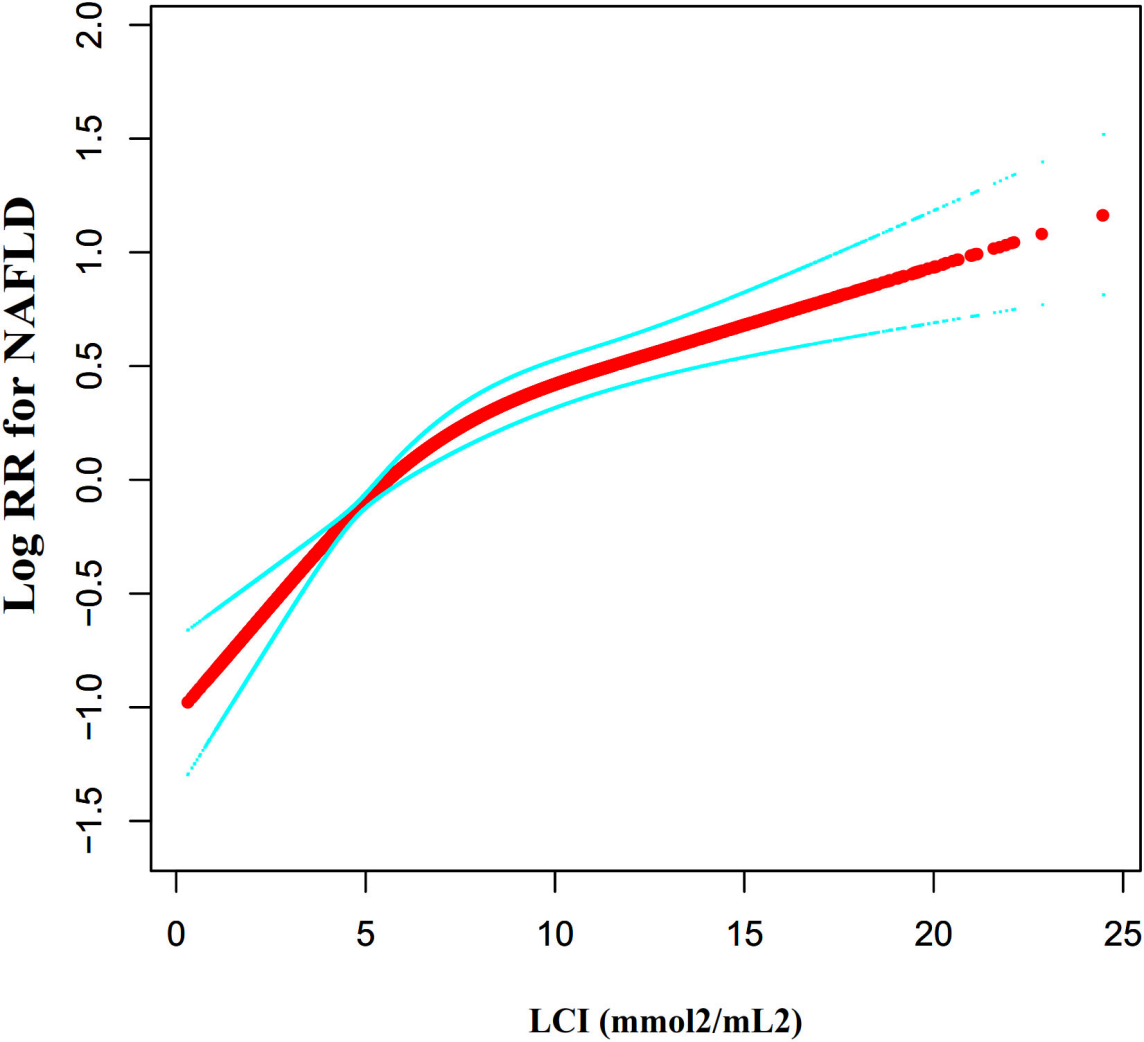
The non-linear relationship between LCI and the risk of NAFLD. We used a Cox proportional hazards regression model with cubic spline functions to evaluate the relationship between LCI and NAFLD risk. The result showed that the relationship between LCI and NAFLD was non-linear, with the inflection point of LCI being 5.514 mmol^2^/L^2^. LCI, lipoprotein combine index; NAFLD, non-alcoholic fatty liver disease.

Figure 7 investigated the correlation between LCI and NAFLD using Cox proportional hazards regression model with cubic spline functions. After adjusting for multiple confounding factors, a significant non-linear relationship was confirmed (log-likelihood ratio test *P* < 0.001). The LCI inflection point was identified at 5.514 mmol^2^/L^2^. Using a two-piecewise Cox proportional-hazards regression model, the hazard ratios were: left of inflection point HR = 1.282 (95% CI: 1.162–1.415), right of inflection point HR = 1.063 (95% CI: 1.042–1.084) (Table 5).

**TABLE 5:** The result of the two-piecewise Cox regression model.

Incident NAFLD	HR,95%CI	*P*
Fitting model by standard Cox regression	1.085 (1.068, 1.102)	<0.0001
Fitting model by two-piecewise Cox regression		
Inflection point of LCI	5.514	103.117
≤ Inflection point	1.282 (1.162, 1.415)	<0.0001
> Inflection point, ≤ 130	1.063 (1.042, 1.084)	<0.0001
P for log-likelihood ratio test		<0.001

We adjusted age, SBP, sex, ALT, BMI, GGT, DBP, ALP, ALB, GLB, DBIL, AST, TB, UA, FBG, BUN. HR, Hazard ratios; CI, confidence; Ref, reference; LCI, lipoprotein combine index (mmol^2^/mL^2^); NAFLD, non-alcoholic fatty liver disease.

### Subgroup analyses

**FIGURE 8:**
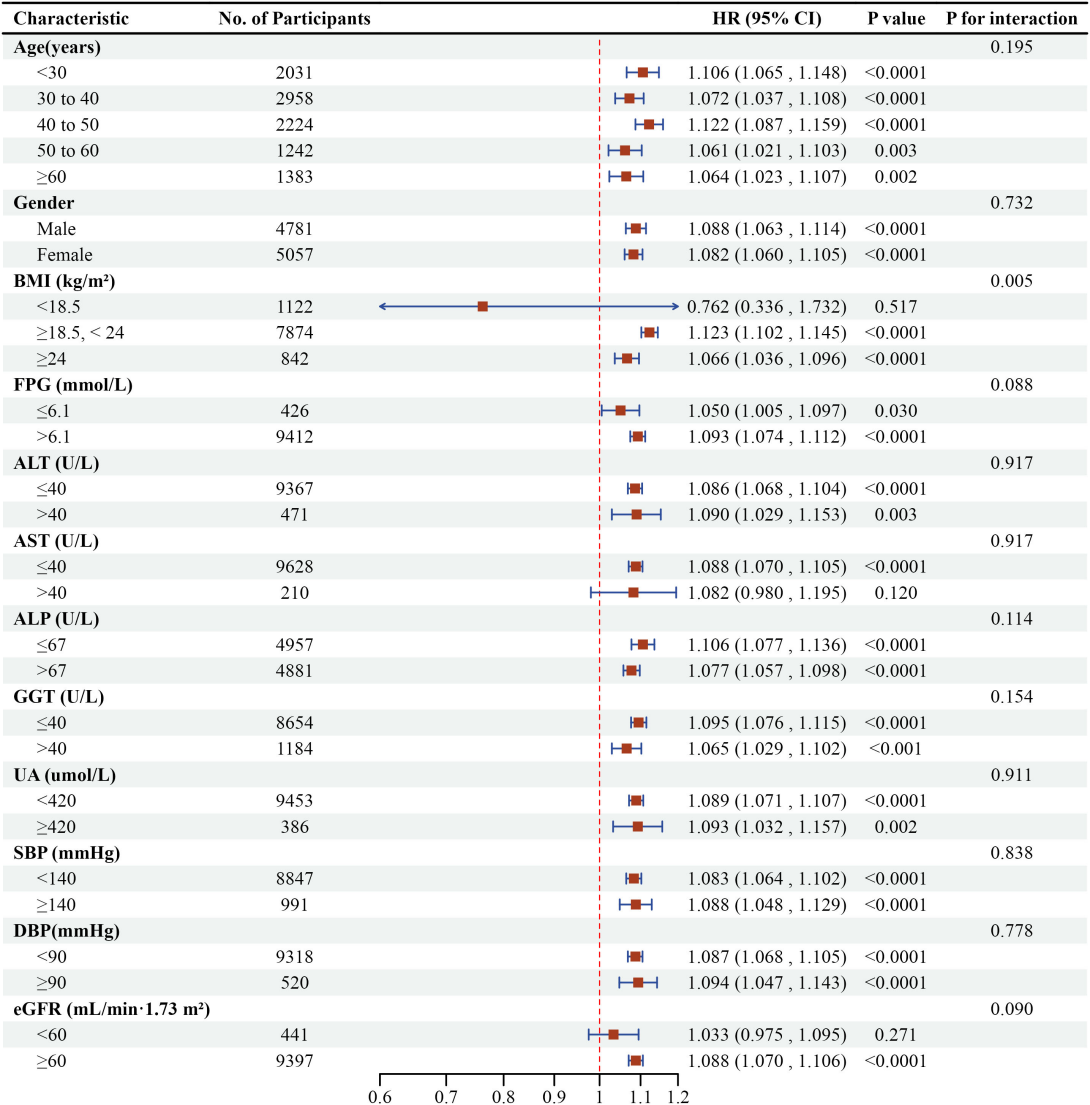
Effect size of LCI on NAFLD in prespecified and exploratory subgroups. Forest plot displaying the association between LCI and NAFLD risk across various subgroups using multivariable Cox proportional hazards regression, with HRs and 95% CIs per unit increase in LCI. A significant interaction was observed for BMI (*P* for interaction < 0.05), with the strongest association found in participants with normal BMI (18.5–24 kg/m^2^).

Subgroup analyses revealed no significant interactions for age, gender, FBG, ALT, AST, ALP, GGT, UA, DBP, eGFR, or SBP (Figure 8). A significant interaction was detected in BMI: individuals with BMI 18.5–24 kg/m^2^ showed the strongest association between LCI and NAFLD (HR = 1.123, 95% CI: 1.102–1.145, *P* < 0.0001). In contrast, those with BMI ≥ 24 kg/m^2^ (HR = 1.066, 95% CI: 1.036–1.096, *P* < 0.0001) and BMI < 18.5 kg/m^2^ (HR = 0.762, 95% CI: 0.336–1.732, *P* = 0.5171) demonstrated weaker associations.

### NAFLD prediction using LCI

**FIGURE 9:**
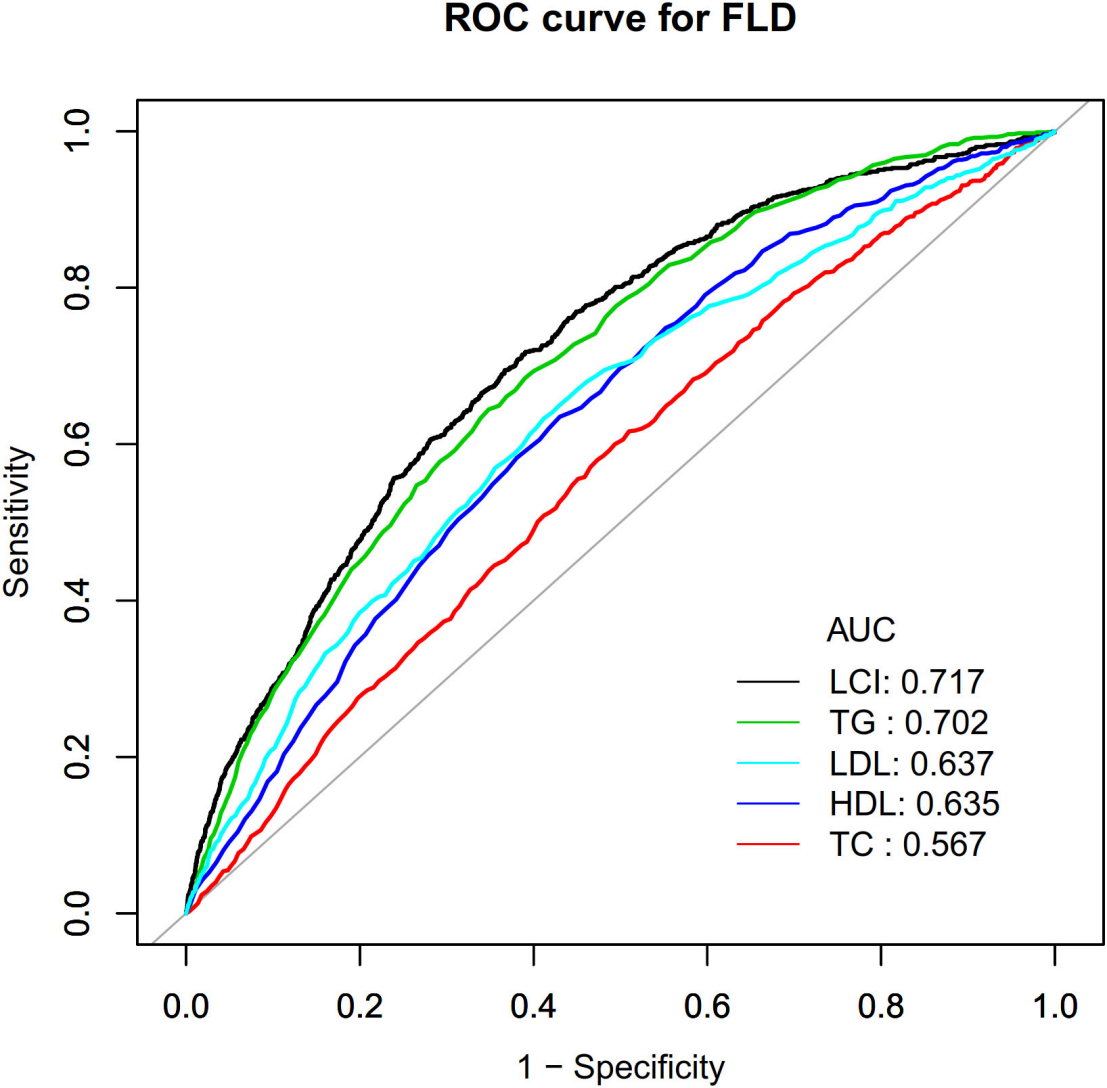
The LCI, TC, TG, HDL-c and LDL-c for predicting NAFLD in all participants by ROC analyses. ROC curves comparing the predictive ability of LCI versus traditional lipid parameters for NAFLD, with LCI demonstrating the highest area under the curve (AUC = 0.717). LCI, lipoprotein combine index; NAFLD, non-alcoholic fatty liver disease; AUC, area under the curve; ROC, receiver operating characteristic.

Receiver operating characteristic (ROC) curve analysis evaluated the predictive capacity of LCI, TC, TG, HDL-c, and LDL-c for NAFLD risk (Figure 9). Areas under the curves were: LCI: 0.717, TC: 0.567, TG: 0.702, HDL-c: 0.636, LDL-c: 0.637. Optimal cut-off values were: LCI: 6.734, TC: 4.365, TG: 1.045, HDL-c: 1.435, LDL-c: 2.205. DeLong methodology confirmed statistically significant differences (*P* < 0.05). LCI demonstrated superior predictive performance, with the highest Youden index and AUC in Table 6.

**TABLE 6:** AUC for each evaluated parameter in identifying NAFLD.

Test	AUC	95% CI	Best threshold	Specificity	Sensitivity	Youden index
LCI	0.717	0.700–0.735	6.734	0.633	0.695	0.328
TC	0.567	0.547–0.586	4.365	0.490	0.616	0.107
TG	0.702	0.685–0.720	1.045	0.652	0.644	0.296
HDL-c	0.636	0.617–0.654	1.435	0.570	0.635	0.205
LDL-c	0.637	0.618–0.657	2.205	0.588	0.633	0.221

CI: confidence; LCI, lipoprotein combine index (mmol^2^/mL^2^); NAFLD, non-alcoholic fatty liver disease; TC, Total cholesterol; TG, Triglyceride; LDL-c, low-density lipid cholesterol; HDL-c, high-density lipoprotein cholesterol; AUC, area under the curve.

## Discussion

In this retrospective cohort study, we discovered that elevated LCI was significantly linked with increased risk of NAFLD development in non-obese Chinese population with normative lipid levels. The association between LCI and NAFLD incidence exhibited a non-linear pattern, with critical turning point at 5.514 mmol^2^/L^2^, where the HR was significantly higher below this threshold (HR = 1.282, 95%CI: 1.162–1.415) compared to above it (HR = 1.063, 95%CI: 1.042–1.084). BMI significantly modified this relationship, with stronger associations observed in normal-weight individuals than lean participants. Additionally, LCI demonstrated a higher predictive capacity for NAFLD than individual lipid parameters with an AUC of 0.717.

This study revealed a cumulative NAFLD occurrence of 8.69% during a median follow-up of 33.10 months, corresponding to an incidence of 31.51 cases per 1000 person-years. This rate varied substantially across LCI quartiles, from 8.68 in the lowest quartile to 66.99 per 1000 person-years in the highest quartile. While broadly consistent with established Asian population studies, our data reveal a nuanced reduction in incidence rates. The meta-analysis conducted by Ye et al. substantiated annual NAFLD prevalence in Asian populations between 35.0 and 52.34 cases per 1000 person-years (5). This relatively lower occurrence rate in our cohort likely reflects our specific inclusion criteria focusing on non-obese individuals with normal lipids profile, which included populations that are often overlooked and typically considered to have relatively low risk (9).

Our Cox regression analysis revealed a significant positive correlation between LCI and NAFLD risk, with each unit increase in LCI corresponding to an 8.5% increased risk of NAFLD after adjusting for confounders. Our results align with the pioneering cross-sectional research by Qiu et al., who initially characterized LCI as a potential NAFLD indicator within a Japanese population (16). However, our study extends these findings by providing longitudinal evidence of this association, establishing the temporal relationship between baseline LCI and subsequent NAFLD development. Additionally, while Qiu et al. focused on NAFLD detection in a general population, our study specifically examined the predictive value of LCI for NAFLD incidence in non-obese individuals with normal blood lipids levels, a population often considered lower risk but increasingly recognized as susceptible to “lean NAFLD”, defined as the presence of hepatic steatosis in individuals with BMI < 25 kg/m^2^ in non-Asian populations or <23 kg/m^2^ in Asian populations without excessive alcohol consumption or other secondary causes of fatty liver (3, 5, 31).

The underlying mechanisms linking LCI to NAFLD development are multifaceted. As a composite index incorporating multiple lipid parameters, LCI reflects a comprehensive assessment that outperforms individual lipid markers in predicting NAFLD development. This superiority stems from LCI’s ability to simultaneously capture the comprehensive engagement of pro-atherogenic and anti-atherogenic components involved in hepatic lipid accumulation (32). The association between LCI and NAFLD can be explained through three interconnected biological mechanisms that directly address hepatic steatosis development. (1) Lipid Metabolism Dysregulation: Elevated TC, TG and LDL-c levels, which increase LCI, promote hepatic lipid accumulation through increased free fatty acid flux and *de novo* lipogenesis (33, 34). Each component of LCI reflects specific aspects of disrupted lipid homeostasis. Elevated TC and LDL-c indicate impaired cholesterol clearance and increased hepatic cholesterol content, which activates sterol regulatory element-binding protein-1c (SREBP-1c) and enhances lipogenic gene expression (35). Elevated TG reflects increased *de novo* lipogenesis and impaired fatty acid oxidation, while reduced HDL-c indicates compromised reverse cholesterol transport and reduced cholesterol efflux from hepatocytes (36). The multiplicative formula of LCI captures the compounding effect when multiple lipid abnormalities occur simultaneously. Reduced HDL-C, also reflected in higher LCI, impairs reverse cholesterol transport and reduces anti-inflammatory protection (37, 38). (2) Liver Fat Accumulation Mechanisms: Additionally, dyslipidemia often coexists with insulin resistance, which further promotes liver lipid accumulation by increasing lipid mobilization in adipose tissue and upregulating lipogenic transcription factors such as SREBP-1c in hepatocytes. Mechanistically, elevated LCI reflects increased substrate availability for hepatic steatosis through two major pathways: enhanced delivery of free fatty acids to the liver and upregulated *de novo* lipogenesis (39). Insulin resistance serves as a central mediator in this process, promoting adipose tissue lipolysis, impairing very low-density lipoprotein secretion, and activating lipogenic transcription factors such as SREBP-1c in hepatocytes (40). (3) Inflammatory Pathways: The lipid abnormalities captured by LCI trigger hepatic inflammatory responses through multiple pathways. Elevated LDL-c promotes oxidative stress and pro-inflammatory cytokine production in hepatocytes, while reduced HDL-c diminishes anti-inflammatory protection and impairs the liver’s ability to resolve inflammation (41). This inflammatory environment further exacerbates insulin resistance and promotes the progression from simple steatosis to more severe forms of NAFLD (32). As LCI comprehensively captures the complex interplay among insulin resistance, lipid metabolism dysregulation, and inflammatory pathways, it more accurately represents the “multiple-hit” pathogenesis of NAFLD compared to isolated lipid measurements (1, 32). These three mechanisms work synergistically to promote NAFLD development, which may help explain why LCI, as a composite index incorporating multiple lipid parameters, shows enhanced predictive performance compared to individual lipid markers in our study.

Statins, as HMG-CoA reductase inhibitors, directly target the lipids components reflected in elevated LCI scores. By reducing hepatic cholesterol synthesis, statins lower circulating LDL-C and may modulate hepatic lipid metabolism pathways implicated in NAFLD pathogenesis (42). Beyond their lipid-lowering effects, statins can mitigate insulin resistance, oxidative stress and fibrogenesis, potentially interrupting the pathophysiological mechanisms linking elevated LCI to NAFLD development (43, 44). These pleiotropic effects may portend statin therapy’s potential in ameliorating hepatic steatosis, particularly in patients with high LCI scores.

Our subgroup investigations uncovered BMI’s potential role as a significant effect modifier in the LCI and NAFLD risk relationship. The most robust statistical correlations emerged among participants categorized in the intermediate BMI spectrum (BMI ≥ 18.5, < 24 kg/m^2^). In contrast, individuals with BMI over 24 kg/m^2^ exhibited considerably weaker associations, while participants with BMI below 18.5 kg/m^2^ showed no significant associations. Whereas previous studies demonstrated that lipid parameters mediate the relationship between NAFLD risk and BMI (45), our research is the first to unveil the adjusting effect of BMI on the correlation between LCI and NAFLD incidence. Mechanistically, previous researches have indicated that higher BMI levels are linked to lower NAFLD risk (2), which explains why higher BMI (≥ 24 kg/m^2^) may partially mask LCI effect. In contrast, lean individuals typically have lower insulin resistance and inflammation levels (31), which may weaken LCI’s predictive value for NAFLD risk. This finding has important clinical implications: it reveals the interaction between LCI and BMI in NAFLD risk, suggesting that LCI has important predictive value for NAFLD incidence in individuals with normal weight and normal blood lipids, providing new insights for individualized NAFLD risk assessment and intervention.

A novel finding in this research is the non-linear relationship between LCI and NAFLD risk, with an inflection point at 5.514 mmol^2^/L^2^. Below this threshold, the association between LCI and NAFLD was substantially stronger compared to values above this point. While no previous studies have specifically examined non-linearity in the LCI-NAFLD relationship, our results are consistent with the work of Chen et al., which demonstrated non-linear relationships between traditional lipid parameters and NAFLD (46). The identified inflection point at LCI = 5.514 mmol^2^/L^2^ represents a critical metabolic threshold with important clinical implications. Below this threshold, LCI demonstrates substantially stronger association with NAFLD development (HR = 1.282, 95%CI: 1.162–1.415) compared to values above this point (HR = 1.063, 95%CI: 1.042–1.084), potentially suggesting distinct metabolic phenotypes (47). Supplementary Table 2 reveals that participants with an LCI of ≤5.514 mmol^2^/L^2^ had a smaller proportion of females and exhibited lower levels of BMI, SBP, DBP, AST, ALP, GGT, UA, and LDL-C. These variables are strongly linked to NAFLD. In this metabolically healthier population, the relative absence of conventional risk factors amplifies the predictive impact of subtle lipid abnormalities captured by LCI (48). This phenomenon aligns with the concept that early metabolic dysfunction may be more readily detected through composite lipid indices before overt clinical manifestations appear (49). When LCI exceeded 5.514 mmol^2^/L^2^, its influence on NAFLD was small. In contrast, below 5.514 mmol^2^/L^2^, these risk contributors for NAFLD were diminished and had a lesser impact, making the LCI effect more pronounced. This curvilinear relationship between LCI and NAFLD has important clinical meaning. This threshold enables enhanced risk stratification, particularly in seemingly low-risk populations. For individuals with LCI below 5.514 mmol^2^/L^2^, targeted lipid-modifying interventions may yield greater NAFLD prevention benefits due to the steeper risk gradient in this range (50). The stronger predictive value in metabolically healthier individuals supports LCI’s utility as a screening tool for populations who might be overlooked by traditional risk assessment strategies. By precisely intervening to reduce LCI below 5.514 mmol^2^/L^2^ in non-obese populations, we can significantly lower the risk of NAFLD. When LCI below this critical point, the incidence of NAFLD events demonstrates a more rapid decline. This research offers an innovative risk assessment and prevention decision-making tool for clinicians.

The AUC value of 0.717 for LCI indicates acceptable discriminatory ability for NAFLD screening (51). A study by Qiu et al. using the NAGALA cohort reported a higher AUC of 0.8118 for LCI in the general population (16). The difference likely reflects distinct study designs (longitudinal cohort vs. cross-sectional) and population characteristics: our study focused on non-obese individuals with normal lipid profiles, while NAGALA included the general population. NAFLD prediction becomes particularly challenging in non-obese, normal-lipid populations where traditional risk factors show limited discriminatory power. Importantly, DeLong test results in both studies confirmed that LCI significantly outperformed individual lipid markers (all *P* < 0.05), validating the superior diagnostic value of composite indices across different populations. For screening applications, moderate discriminatory ability remains clinically meaningful when identifying individuals requiring further evaluation (52). The value of LCI lies in its practical advantages as a cost-effective, non-invasive tool that enhances NAFLD risk stratification without additional laboratory costs.

This study has several notable advantages: (1) We employed a substantial sample size and ensured adequate follow-up, establishing the temporal relationship between baseline LCI and subsequent NAFLD development. (2) Significantly, this study is the first to thoroughly investigate the predictive capacity of LCI for NAFLD in non-obese individuals who have normal blood lipids, an often-overlooked population. (3) Rigorous statistical adjustments were implemented to reduce possible confounding factors. (4) Our comprehensive statistical approach, including non-linear modeling, provided nuanced insights into the LCI-NAFLD relationship. (5) The robustness of our findings was assessed using a range of stratified analyses and sensitivity testing approaches.

Despite these strengths, several limitations should be acknowledged. First, the diagnosis of NAFLD relied on ultrasonography instead of liver biopsy, limiting assessment of disease severity. Second, our study included exclusively Chinese individuals, potentially limiting generalizability to other populations. Third, we only assessed baseline LCI without accounting for changes over time. Finally, our study used NAFLD rather than the recently proposed metabolic dysfunction-associated fatty liver disease (MAFLD) classification, as our design predated this change. Nevertheless, epidemiological data suggest more than 95% of NAFLD patients fulfill the criteria for MAFLD, supporting that our findings can likely be extrapolated to the MAFLD framework (53).

## Conclusion

In conclusion, our study demonstrates a positive and non-linear association between LCI and NAFLD risk in non-obese Chinese individuals with normal lipids. While LCI showed a relative advantage over individual lipid parameters in NAFLD prediction, the moderate discriminatory ability (AUC = 0.717) suggests its utility may be primarily as a practical screening marker in normal-weight individuals. Our analysis revealed this relationship exhibits a threshold effect, where NAFLD risk increases significantly when the LCI falls below 5.514 mmol^2^/L^2^. These findings suggest that LCI assessment may serve as a complementary screening approach for identifying individuals at risk in seemingly low-risk populations with normal lipid profiles. However, given the moderate predictive performance, clinical implementation should be considered cautiously and in conjunction with other risk factors. Future studies should validate these findings across diverse populations and investigate whether LCI-guided screening strategies can effectively contribute to early NAFLD detection and prevention efforts.

## Ethical approval and consent to participate

As previously reported, the study was conducted in strict adherence to the ethical guidelines of the Declaration of Helsinki (10). Each participant provided oral informed consent prior to study involvement. Personal identifiable information was anonymized and replaced with a medical examination identifier. The Ethics Committee of Wenzhou People’s Hospital reviewed and approved the research protocol (10).

## Data Availability

The raw data supporting the conclusions of this article will be made available by the authors, without undue reservation.

## Footnotes

1
www.datadryad.org

2
http://www.R-project.org

3
http://www.empowerstats.com
