# Supplementary material for: Lipoprotein combine index is associated with non-alcoholic fatty liver disease: a 5-year longitudinal cohort study in non-obese Chinese populations with normal lipids
Source: Front Med (Lausanne). 2025 Aug 15;12:1618576. doi: 10.3389/fmed.2025.1618576 (PMC12394169; doi:10.3389/fmed.2025.1618576)
Supplement: Supplementary file 1 [file Data_Sheet_1.pdf]

Table S1. Collinearity diagnostics steps.

Variable	VIF<br>Step 1
**Gender**	1
**Age (years)**	1.1
**ALP (U/L)**	1.2
**GGT (U/L)**	1.3
**ALT (U/L)**	3
**AST (U/L)**	3.1
**ALB (g/L)**	1.2
**GLB (g/L)**	1.1
**TB (μmol/L)**	2.2
**DBIL (μmol/L)**	2.2
**BUN (mmol/L)**	1.4
**Scr (μmol/L)**	1.5
**UA (μmol/L)**	1.5
**GLU (mmol/L)**	1.2
**BMI (kg/m²)**	1.2
**DBP (mmHg)**	2.2
**SBP (mmHg)**	2.5

VIF: variance inflation factor.

Note: When the Variance Inflation Factor (VIF) exceeds 5, it indicates significant multicollinearity among the variables. To ensure the integrity of regression models, it is essential to remove these variables from the analysis.

BMI, Body mass index; DBP, Diastolic blood pressure; ALP, Alkaline phosphatase; SBP, Systolic blood pressure; GGT, γ-glutamyl transpeptidase; AST, Aspartate aminotransferase; TG, Triglyceride; ALB, albumin; ALT, Alanine aminotransferase; GLB, globulin; LDL-C, Low-density lipid cholesterol; BUN, Serum urea nitrogen; HDL-C, High-density lipoprotein cholesterol; Scr, Serum creatinine; TC, Total cholesterol; FPG, Fasting plasma glucose; UA, uric acid; DBIL, Direct bilirubin; TB, Total bilirubin; LCI, Lipoprotein combine index.

Table S2. The characteristics of participants on both sides of the inflection point.

LCI group (mmol²/L²)	>5.514	≤5.514	P-value
**Participants**	5088	4750	
**Age (years)**	42.91 ± 14.86	41.97 ± 14.52	0.002
**ALP (U/L)**	71.35 ± 21.48	65.48 ± 23.21	<0.001
**ALT (U/L)**	26.66 ± 23.58	20.99 ± 21.66	<0.001
**GGT (U/L)**	16.00 (12.00-23.00)	14.00 (10.00-21.00)	<0.001
**AST (U/L)**	21.00 (18.00-25.00)	20.00 (17.00-25.00)	<0.001
**ALB (g/L)**	44.40 ± 2.75	44.15 ± 2.75	<0.001
**GLB (g/L)**	29.67 ± 3.91	29.25 ± 3.82	<0.001
**TB (μmol/L)**	12.23 ± 4.88	12.08 ± 5.01	0.053
**DBIL (μmol/L)**	2.30 ± 1.20	2.43 ± 1.19	<0.001
**BUN (mmol/L)**	4.51 ± 1.29	4.42 ± 1.39	<0.001
**Scr (μmol/L)**	80.26 ± 23.84	73.59 ± 26.49	
**UA (μmol/L)**	281.01 ± 79.53	245.59 ± 76.19	<0.001
**FPG (mmol/L)**	5.15 ± 0.74	4.99 ± 0.65	<0.001
**BMI (kg/m²)**	21.53 ± 1.97	20.49 ± 1.98	<0.001
**SBP (mmHg)**	120.75 ± 16.22	115.22 ± 15.46	<0.001
**DBP (mmHg)**	72.99 ± 10.08	69.49 ± 9.50	<0.001
**Gender**			<0.001
**Female (%)**	2340 (45.99%)	2441 (51.39%)	
**Male (%)**	2748 (54.01%)	2309 (48.61%)	

Values are n (%) or mean ± SD or median (quartile)

BMI, Body mass index; DBP, Diastolic blood pressure; ALP, Alkaline phosphatase; SBP, Systolic blood pressure; GGT, γ-glutamyl transpeptidase; AST, Aspartate aminotransferase; TG, Triglyceride; ALB, albumin; ALT, Alanine aminotransferase; GLB, globulin; LDL-C, Low-density lipid cholesterol; BUN, Serum urea nitrogen; HDL-C, High-density lipoprotein cholesterol; Scr, Serum creatinine; TC, Total cholesterol; FPG, Fasting plasma glucose; UA, uric acid; DBIL, Direct bilirubin; TB, Total bilirubin; LCI, Lipoprotein combine index.

Table S3 Relationship between LCI and the incident NAFLD in pre-imputation data and imputed datasets

Exposure	pre-imputation	imputation 1	imputation 2	imputation 3	imputation 4	imputation 5
**Non-adjusted**						
**LCI**	1.174 (1.157, 1.191) <0.00001	1.174 (1.157, 1.191) <0.00001	1.174 (1.157, 1.191) <0.00001	1.174 (1.157, 1.191) <0.00001	1.174 (1.157, 1.191) <0.00001	1.174 (1.157, 1.191) <0.00001
**LCI Quartile**						
**Q1**	Ref.	Ref.	Ref.	Ref.	Ref.	Ref.
**Q2**	2.091 (1.537, 2.845) <0.00001	2.091 (1.537, 2.845) <0.00001	2.091 (1.537, 2.845) <0.00001	2.091 (1.537, 2.845) <0.00001	2.091 (1.537, 2.845) <0.00001	2.091 (1.537, 2.845) <0.00001
**Q3**	3.765 (2.831, 5.007) <0.00001	3.765 (2.831, 5.007) <0.00001	3.765 (2.831, 5.007) <0.00001	3.765 (2.831, 5.007) <0.00001	3.765 (2.831, 5.007) <0.00001	3.765 (2.831, 5.007) <0.00001
**Q4**	7.597 (5.801, 9.949) <0.00001	7.597 (5.801, 9.949) <0.00001	7.597 (5.801, 9.949) <0.00001	7.597 (5.801, 9.949) <0.00001	7.597 (5.801, 9.949) <0.00001	7.597 (5.801, 9.949) <0.00001
**P for trend**	<0.00001	<0.00001	<0.00001	<0.00001	<0.00001	<0.00001
**Adjust I**						
**LCI**	1.107 (1.090, 1.124) <0.00001	1.107 (1.090, 1.124) <0.00001	1.107 (1.090, 1.124) <0.00001	1.107 (1.090, 1.124) <0.00001	1.107 (1.090, 1.124) <0.00001	1.107 (1.090, 1.124) <0.00001
**LCI Quartile**						
**Q1**	Ref.	Ref.	Ref.	Ref.	Ref.	Ref.
**Q2**	1.737 (1.277, 2.365) 0.00044	1.737 (1.277, 2.364) 0.00044	1.737 (1.277, 2.365) 0.00044	1.737 (1.277, 2.365) 0.00044	1.737 (1.277, 2.365) 0.00044	1.737 (1.277, 2.365) 0.00044
**Q3**	2.396 (1.799, 3.191) <0.00001	2.393 (1.797, 3.188) <0.00001	2.394 (1.797, 3.188) <0.00001	2.393 (1.797, 3.187) <0.00001	2.393 (1.797, 3.187) <0.00001	2.393 (1.797, 3.188) <0.00001
**Q4**	3.721 (2.833, 4.887) <0.00001	3.720 (2.832, 4.885) <0.00001	3.720 (2.832, 4.886) <0.00001	3.720 (2.833, 4.886) <0.00001	3.720 (2.833, 4.886) <0.00001	3.720 (2.832, 4.885) <0.00001
**P for trend**	<0.00001	<0.00001	<0.00001	<0.00001	<0.00001	<0.00001
**Adjust II**						
**LCI**	1.077 (1.053, 1.101) <0.00001	1.081 (1.064, 1.099) <0.00001	1.086 (1.069, 1.103) <0.00001	1.089 (1.072, 1.106) <0.00001	1.086 (1.068, 1.103) <0.00001	1.085 (1.068, 1.102) <0.00001
**LCI Quartile**						
**Q1**	Ref.	Ref.	Ref.	Ref.	Ref.	Ref.
**Q2**	1.555 (1.016, 2.378) 0.04192	1.661 (1.218, 2.265) 0.00135	1.601 (1.174, 2.182) 0.00291	1.612 (1.182, 2.199) 0.00256	1.633 (1.198, 2.227) 0.00193	1.672 (1.226, 2.282) 0.00118
**Q3**	2.129 (1.431, 3.166) 0.00019	2.153 (1.611, 2.878) <0.00001	2.232 (1.672, 2.979) <0.00001	2.109 (1.578, 2.819) <0.00001	2.198 (1.646, 2.935) <0.00001	2.209 (1.652, 2.954) <0.00001
**Q4**	2.590 (1.769, 3.791) <0.00001	2.940 (2.226, 3.882) <0.00001	3.026 (2.293, 3.993) <0.00001	2.991 (2.265, 3.949) <0.00001	3.005 (2.276, 3.967) <0.00001	3.022 (2.288, 3.991) <0.00001
**P for trend**	<0.00001	<0.00001	<0.00001	<0.00001	<0.00001	<0.00001

HR (95% CI) P value

Crude model: we did not adjust other covariates.

Model I: we adjusted age, sex, BMI, SBP, DBP.

Model II: we adjusted age, SBP, sex, ALT, BMI, GGT, DBP, ALP, ALB, GLB, DBIL, AST, TB, UA, FBG and BUN.

CI: confidence interval, Ref: reference.
